# Clinical Holistic Medicine: Holistic Treatment of Children

**DOI:** 10.1100/tsw.2004.116

**Published:** 2004-08-04

**Authors:** Søren Ventegodt, Mohammed Morad, Gideon Vardi, Joav Merrick

**Affiliations:** ^1^ The Quality of Life Research Center, Teglgårdstræde 4-8, DK-1452 Copenhagen K, Denmark; ^2^ Division of Community Health, Ben Gurion University, Beer-Sheva, Israel; ^3^ Zusman Child Development Center, Division of Pediatrics, Soroka University Medical Center, Beer-Sheva, Israel; ^4^ National Institute of Child Health and Human Development, Office of the Medical Director, Division for Mental Retardation, Ministry of Social Affairs, Jerusalem and Zusman Child Development Center, Division of Pediatrics and Community Health, Ben Gurion University, Beer-Sheva, Israel

**Keywords:** quality of life, QOL, philosophy, human development, holistic medicine, public health, holistic health, holistic process theory, life mission theory, group therapy, Denmark

## Abstract

We believe a holistic approach to problems in childhood and adolescence will benefit the child, adolescent, and the whole family. As a rule, children have far less to say in the family than their parents. Therefore, it is the parents who set the agenda and decide how things are done at home and in relation to the child. Most often, it is also the parents who have a problem when the child is not thriving. The child thus acts as the thermometer of the family. When children are not feeling well or are sick, the parents are not doing well either. Most problems arising from dysfunctional patterns are almost impossible for the parents to solve on their own, but with help and support from the holistically oriented physician, we believe that many problems can be discovered and solved. Not only can health problems be addressed, but also problems of poor thriving in the family in general. With the physician in the role of a coach, the family can be provided with relevant exercises that will change the patterns of dysfunction. Consciousness-based medicine also seems to be efficient with children and adolescents, who are much more sensitive to the psychosocial dimensions than adults. Five needs seem to be essential for the thriving and health of the child: attention, respect, love, acceptance (touch), and acknowledgment. The physician should be able to see if the child lacks fulfillment in one or more of these needs, and he can then demonstrate to the parents how these needs should be handled. This should be followed by simple instructions and exercises for the parents in the spirit of coaching. This approach is especially relevant when the child is chronically ill.

## INTRODUCTION

Sometimes you will hear the following sentence: "If your child is impossible for you, you are also impossible for your child." A number of recent studies have shown that around one in four children in Denmark have social, psychological, or health problems[1,2,3]. Some of these studies were conducted by Professor Per Schultz Jørgensen, from the Royal Danish School of Educational Studies, who also chaired the Danish National Council for Children (Children Ombudsman) during 1998–2001 and these studies make sad and dismal reading. We find it even more upsetting that the number of children and families in distress does not appear to be changing, despite the fact that increasing attention has been given to the problem.

When children are not feeling well psychosocially, it can be manifested by a range of symptoms such as frequent bedwetting, stomachache, sleep problems, disruption at school, bullying, problems with learning, or concentration. The parents may also find their children to be completely impossible, nonreceptive to common sense or any reasonable attempt at upbringing. The physician will often find it impossible to treat these symptoms with biomedicine. We generally do not like giving children drugs that suppress symptoms because of the long-term consequences. Our holistic approach to children is not, in principal, different from our holistic approach to adult patients except that psychosocial factors seem to be of even more importance with children. Let us therefore first take a short review of our work relevant for holistic medicine in general.

## THE FOUNDATION OF CLINICAL HOLISTIC MEDICINE

The life mission theory[4,5,6,7,8,9] states that everybody has a purpose of life or a huge talent. Happiness comes from living this purpose and succeeding in expressing the core talent in your life. To do this, it is important to develop as a person into what is known as the natural condition, a condition where you know yourself and use all your efforts to achieve what is most important for you. The holistic process theory of healing[10,11,12,13] and the related quality of life theories[14,15,16] state that the return to the natural state of being is possible whenever the person gets the resources needed for existential healing. The resources needed are "holding" in the dimensions awareness, respect, care, acknowledgment, and acceptance with support and processing in the dimensions feeling, understanding, and letting go of negative attitudes and beliefs. The preconditions for holistic healing to take place are trust and the intention of the healing to take place. Existential healing is not a local healing of any tissue, but a healing of the wholeness of the person, making him much more resourceful, loving, and knowledgeable of himself, his own needs and wishes. In letting go of negative attitudes and beliefs, the person returns to a more responsible existential position and an improved quality of life. The philosophical change in the person healing is often a change towards preferring difficult problems and challenges instead of avoiding difficulties in life[17,18,19,20,21,22,23,24]. The person who becomes happier and more resourceful often also becomes more healthy, more talented, and more able to function[25,26,27].

## THE CHILD AS THE THERMOMETER OF THE FAMILY

It is of great advantage to be able to adopt a holistic perspective in relation to the child. If it is possible to assess and observe the whole family and not just the child, it is often possible to understand the interaction and where the problem arises. As a rule, children have far less say in the family than their parents with the parents setting the agenda and deciding how things are done at home and in relation to the child. It is also, therefore, most often the parents who have a problem when the children are impossible. You can say that the children function as the thermometer of the family. When children are ill or not doing well, this is often due to the parents not doing well either or unable to cope with themselves, with each other, and with the children.

## CHILDREN COOPERATE

It is a natural reaction to blame impossible children for their impossibility. But, as the family therapist Jesper Juul, the founder of the Scandinavian Kempler Institute in Denmark, has so outstandingly explained in his book *Your Competent Child*[28], children like to cooperate and they will do whatever they can to be loved by their parents. But children have their needs, which they are compelled to fight for in order to have fulfilled, even if this cuts across their parents. The following things in particular are concerned:

- The need for attention: being seen and understood
- The need for respect: being met and acknowledged as an independent living being
- The need for love: care, loving touch, and loving words.
- The need for touch and acceptance
- The need for acknowledgment: the parents must see and fully acknowledge the character of body, mind, and spirit

Instead, children are often given the brush-off, met with emotional coldness, or perhaps even punished with studied indifference or in some other way by their frustrated parents. When parents and children get on well together, the child is the source of the greatest joy in the world. Many modern parents appreciate their children, are very attentive to their children, and are very aware of the relationship, but they nevertheless often overlook a number of serious imbalances. The child may be suffocated by *too much* care and protection or may become domineering, even a complete prima donna, from receiving too much attention or attention of a neurotic and idolizing nature. The child might even become domineering and take up too much space from misunderstood respect of boundaries and independence. It is more usual, however, for the children to be neglected, ignored, insulted, scolded, and condemned.

It is not easy to be a child, but it is just as true to say that it is not easy to be a parent. A good holistic cure for the child, therefore, is support for the parents in being good parents with an understanding of the needs of their children. The project is not concerned with the child, but with the parents and how they deal with the child. As they are the adults, they must also take responsibility and stop being impossible for the child, because when they do, the child will start to do well and if he or she is sick, to get better. And soon the child will flourish and be a joy to the parents, which they had never thought to be possible.

> **Male, age 1 year, and neglect:** 12-month well-baby examination in the family practice with vaccination. Seems a little neglected. The mother also has another young child in her care, who is a late developer and to whom she often gives priority. There is clear jealousy and demand for attention, which triggers scolding. Ears: nothing abnormal discovered. Talk about it. Recommended reading: Jesper Juul, *Your Competent Child*[28].

It is upsetting that so many small children are "maltreated" in Denmark. Physical violence is prohibited, but neglect and other forms of abuse are "permitted". At the well-baby visits, it also seems that quite a number of mothers do not even know what they are doing when they scold a 1-year-old child. Criticism is experienced by the child as if the "King" himself told him and can be experienced as blows, just as bad as if they had been physical blows; indeed, perhaps worse. In our clinical life, we try to use any opportunity to teach parents about the need for care, attention, and respect for children. But parents, who themselves have "been failed" in their childhood, must face up to their failures and old wounds in order to be able to act better towards their own children. This is very painful and we proceed very slowly with them. A good book, which they can take at their own pace to read, is a good idea.

> **Male, aged 3 years, and agitated:** 3-year development examination in the family practice. Good development, he has very good motor skills, but his language skills are less well developed, he is unable or unwilling to repeat syllables. Not so cooperative in

> the vision test, which will have to be carried out in a later examination. Some problems in day care, where the teacher finds him too agitated. Here he tears up the paper on the couch. Perhaps he needs to be met at his boundary [i.e., in balanced contact with his mother, when she crosses the boundary her son has dominated] more than the mother is doing at present. We talk briefly about this.

A 3-year-old boy who acts in protest would seem to have been browbeaten rather than met. He “fights for his life” everywhere, including when he is brought to see the family physician. There is no reason to be angry with him. On the contrary, it is fine that he fights. As a holistic physician, one has “a bone to pick” with the mother, to whom the problem and its possible causes must be discreetly turned over. If she makes changes and meets him at his boundary, their life together will be completely transformed, into fun and games instead of war and argument, from pain to joy for them both in the years to come. Think what a difference such a small change from battling with the boy to showing him respect will make!

> **Female, aged 5 years, with problems concerning respect:** 5-year examination – development good, but there are probably some problems with being respected. Has a great need to take care of herself. Does not want to be vaccinated, and I do not wish to enforce it. Is to come back in 3 months for diphtheria-tetanus vaccination. Social: Today appears shy and inhibited. It appears as though the father violates the patient’s boundaries. Says in as many words that he has to put her in her place the whole time. He regards her as impossible and has some problems in bringing her up. He gets on better with his son. Discussion on children’s needs: care, attention, and respect. I am not sure that he understood. They are to come back in 3 months for a further attempt at vaccination. This is a good opportunity to return to the subject.

In this case, the needs of the girl were discussed with the father who seemed to have difficulties providing care and respect because of lack of knowledge. Our function as physician is not to become emotional and condemnatory, but to remain neutral and helpful. The lack of self-respect in the father, which deep down is what it is about, is a widespread condition and the father needs our respect, not our disrespect. If we are to break the social heritage, we need to make him understand what is going on. If he does understand, a long and difficult process will be started in him where he gradually takes responsibility for all the pain there is in him from long ago in his own childhood, which having a young child constantly reminds him of. The alternative is for him to continue to take out his frustration on his child, become angry with her and irritated by her instead of taking responsibility for the pain in himself. The child is blamed for the discomfort he feels (that is, the discomfort he felt when he himself was a child).

> **Female, aged 3 years, and afraid of animals (arachnophobia):** Increasingly afraid of small animals over the course of a year – she now screams and becomes completely hysterical as soon as she sees a small animal – she says herself that she is most afraid of spiders. Father and mother immediately kill all the small animals they can find and equipped the girl with a fly-swatter so that she can defend herself. The father, who comes with his daughter, also says that he really cannot stand seeing the patient become so frightened. I ask the father whether it is OK for his daughter to become afraid and we are in agreement that it is. She is allowed to have the feelings she has. So he must just hold onto her and support her as well as he can in the meantime. I capture a small animal outside and show it to the patient, while the father holds onto her, and she is not afraid. It appears as though the pattern has been broken. They are to come back if the problem persists.

To us, it is great medical art, although it is extremely simple: The girl did not receive support from her father when she was afraid. On the contrary, he reacted inappropriately (in a neurotic manner) and in so doing reinforced the girl's anxiety. From our point of view, not only did he reinforce her anxiety, but it is actually the disturbance in him that has made the girl afraid of animals in the first place. So in reality, it is the father we are treating here under the disguise of attending to the girl. A more difficult, but in the longer term more effective, strategy would be to make the father understand that he is himself disturbed and over-protective.

> **Male, aged 10 years, with pain in the foot due to a "I hide myself" posture:** Walks on the inside of the foot and is in pain after sport – judo and badminton. As a result of a stooping, "I hide myself" posture, the pelvis is tilted and the knees are turned inward, producing functional flat-footedness. On examination: when the knees are bent out over the foot, the flat-footedness is straightened out and the arch is lifted off the floor. The metatarsal arch is flattened. Conclusion: There is no major anatomical defect, the problem is functional. The patient is instructed to lift his knee up over the side of the mid-foot and to "walk like a cowboy". Social: Patient appears to be affected by having moved, at his own request, from his mother to his father and stepmother, with whom he has a very good relationship (she comes along with him). However, he still sees his mother every other weekend. He is to come back in a month, when we will see whether there has been any improvement following the exercises.

Social and family-related problems hit children and adolescents hard. The defense mechanism is often some negative attitudes in life, which are directly reflected in the way the body is used. Often incorrect use of the body leads, in turn, to problems with the locomotor system as in the case described above. A problem of this kind is difficult to solve with insoles in the shoes, but when the problem is seen in context and thoroughly understood, it is not difficult to do something about it.

> **Female, 39 years, setting boundaries for oneself, not for one's child:** 1. Inflammation particularly in left Achilles tendon. On examination: slight reddening around the skin, resembles streptococcal infection. Prescribe Fucidin cream tid, washing occasionally with soapy water. 2. The problem with regard to setting boundaries in relation to her domineering daughter has improved greatly now that the mother sets boundaries for herself.

The patient presents with a minor physical complaint and we talk about how she can become a better mother for her daughter by setting boundaries for herself (staying in her own area) instead of setting boundaries for her daughter, stamping on her, and trying to solve the problems by subjugating her. Either the daughter can be supported in applying her intelligence and her desire to cooperate or an attempt can be made to condition her — in the same way that Pavlov trained his dogs. The former leads to lively, happy, and healthy children, while the latter results in neurotic children and later in neurotic adults.

## DISCUSSION

Many parents do not understand the ill health of their child as they often have difficulties seeing the child's unfulfilled needs and the dysfunctional patterns in the family. The idea that psychosocial dynamics are a cofactor in the etiology is often strongly provoking, so the perspective must be presented wisely and cautiously to avoid psychological resistance with a poor outcome of holistic therapy.

Many parents hope that the disease of their child will be cured by a simple pharmacological treatment. When the child remains sick, parents will eventually, if guided by a physician proficient in using holistic tools[29], gradually open up to a psychosocial perspective, where the physician can support the development of the quality of life of the child and the quality of life in the whole family. Another problem often seen more and more in the clinic is the holistically oriented parent who does not want to give the children the prescribed drugs. In this case, holistic medicine is an obvious choice, if appropriate in the situation. Sometimes a biomedical treatment is also needed, as in the case where antibiotics are the right choice to cure the child and the role of the physician will be to teach the parents the use and limitations of the different medical paradigms[30], a job that in the end might even save the life of the child.

## CONCLUSION

Many problems noticed in the clinic are almost impossible for the parents to solve on their own, but with a little help from the holistically oriented physician, who understands that the child is the thermometer of the family functioning, many problems can be solved. Not only health problems, but also problems of poor thriving in the family, can be addressed. When the physician takes on a role of coach and provides the family with small relevant exercises, even larger patterns of dysfunction can be turned. Consciousness-based medicine seems to be extremely efficient with children and adolescents, who are much more sensitive to the psychosocial dimensions than adults. Five needs seems to be essential for the thriving and health of the child: attention, respect, love, touch, and acknowledgment[28].

The physician should be able to see if the child lacks fulfillment in one or more of these needs and he can then demonstrate to the parents how these needs should be met. This should be followed by simple instructions and exercises for the parents in the spirit of coaching. This approach is especially relevant when the child is chronically ill.

## ACKNOWLEDGMENTS

This study was supported by grants from IMK Almene Fond. The quality of life research was approved by the Copenhagen Scientific Ethical Committee under number (KF)V.100.2123/91.

## BIOSKETCHES

**Søren Ventegodt, MD,** is the Director of the Quality of Life Research Center in Copenhagen, Denmark. He is also responsible for a Research Clinic for Holistic Medicine in Copenhagen and is a popular speaker throughout Scandinavia. He has published numerous scientific or popular articles and a number of books on holistic medicine, quality of life, and quality of working life. His most important scientific contributions are the comprehensive SEQOL questionnaire, the very short QoL5 questionnaire, the integrated QOL theory, the holistic process theory, the life mission theory, and the Danish Quality of Life Research Survey, 1991–94 in cooperation with the University Hospital of Copenhagen and the late pediatric professor Bengt Zachau-Christiansen. E-mail: ventegodt@livskvalitet.org. Website: http://www.livskvalitet.org

**Mohammed Morad, MD**, is Specialist in Family Medicine, Lecturer in Family Medicine at the National Institute of Child Health and Human Development, Division of Community Health, Ben Gurion University of the Negev and the Medical Director of a large area clinic in the city of Beer-Sheva. He has publications on Bedouin health, health aspects, spiritual health, and aging in persons with intellectual disability, and is a presenter on topics such as health policy and services for the disadvantaged at national and international conferences. E-mail: morad62@barak-online.net

**Gideon Vardi, MD, MPH,** is a specialist in pediatrics, child health, and development working at the Zusman Child Development Center, Division of Pediatrics, Faculty of Health Sciences, Ben Gurion University of the Negev in Beer-Sheva, Israel. His has interest and several publications in the field of child health and human development, rehabilitation, intellectual disability, and disability. E-mail: GideonVa@clalit.org.il

**Joav Merrick, MD, DMSc,** is Professor of Child Health and Human Development affiliated with the Zusman Child Development Center, Division of Pediatrics and Community Health at the Ben Gurion University, Beer-Sheva, Israel; the Medical Director of the Division for Mental Retardation, Ministry of Social Affairs, Jerusalem; and the Founder and Director of the National Institute of Child Health and Human Development. He has numerous publications in the field of child and human development, rehabilitation, intellectual disability, disability, health, welfare, abuse, advocacy, quality of life, and prevention. Dr. Merrick received the Peter Sabroe Child Award for outstanding work on behalf of Danish Children in 1985 and the International LEGO-Prize (“The Children’s Nobel Prize”) for an extraordinary contribution towards improvement in child welfare and well being in 1987. E-mail: jmerrick@internet-zahav.net. Website: www.nichd-israel.com
